# Prediction of velocity profile of water based copper nanofluid in a heated porous tube using CFD and genetic algorithm

**DOI:** 10.1038/s41598-021-90201-x

**Published:** 2021-05-19

**Authors:** Tiziana Ciano, Massimiliano Ferrara, Meisam Babanezhad, Afrasyab Khan, Azam Marjani

**Affiliations:** 1 Department of Law, Economics and Human Sciences & Decisions Lab, Mediterranea University of Reggio Calabria, 89125 Reggio Calabria, Italy; 2 ICRIOS-The Invernizzi Centre for Research in Innovation, Organization, Strategy and Entrepreneurship, Bocconi University-Department of Management and Technology, Via Sarfatti, 25, 20136 Milan, MI Italy; 3 Institute of Research and Development, Duy Tan University, Da Nang, 550000 Vietnam; 4 Faculty of Electrical–Electronic Engineering, Duy Tan University, Da Nang, 550000 Vietnam; 5 Department of Artificial Intelligence, Shunderman Industrial Strategy Co., Tehran, Iran; 6 Institute of Engineering and Technology, Department of Hydraulics and Hydraulic and Pneumatic Systems, South Ural State University, Lenin Prospect 76, Chelyabinsk, 454080 Russian Federation; 7 Department of Chemistry, Islamic Azad University, Arak, Iran

**Keywords:** Chemistry, Energy science and technology, Mathematics and computing, Nanoscience and technology

## Abstract

The heat transfer improvements by simultaneous usage of the nanofluids and metallic porous foams are still an attractive research area. The Computational fluid dynamics (CFD) methods are widely used for thermal and hydrodynamic investigations of the nanofluids flow inside the porous media. Almost all studies dedicated to the accurate prediction of the CFD approach. However, there are not sufficient investigations on the CFD approach optimization. The mesh increment in the CFD approach is one of the challenging concepts especially in turbulent flows and complex geometries. This study, for the first time, introduces a type of artificial intelligence algorithm (AIA) as a supplementary tool for helping the CFD. According to the idea of this study, the CFD simulation is done for a case with low mesh density. The artificial intelligence algorithm uses learns the CFD driven data. After the intelligence achievement, the AIA could predict the fluid parameters for the infinite number of nodes or dense mesh without any limitations. So, there is no need to solve the CFD models for further nodes. This study is specifically focused on the genetic algorithm-based fuzzy inference system (GAFIS) to predict the velocity profile of the water-based copper nanofluid turbulent flow in a porous tube. The most intelligent GAFIS could perform the most accurate prediction of the velocity. Hence, the intelligence of GAFIS is tested for different values of cluster influence range (CIR), squash factor(SF), accept ratio (AR) and reject ratio (RR), the population size (PS), and the percentage of crossover (PC). The maximum coefficient of determination (~ 0.97) was related to the PS of 30, the AR of 0.6, the PC of 0.4, CIR of 0.15, the SF 1.15, and the RR of 0.05. The GAFIS prediction of the fluid velocity was in great agreement with the CFD. In the most intelligent condition, the velocity profile predicted by GAFIS was similar to the CFD. The nodes increment from 537 to 7671 was made by the GAFIS. The new predictions of the GAFIS covered all CFD results.

## Introduction

There are a number of fundamental theories behind the wall heat transfer enhancement. Disturbing the fluid flow, decreasing the thermal boundary layer, changing the fluid thermophysical properties, increasing the heat transfer area, etc. are some of the theories that could be resulted in the increase of the rate of heat transfer in the wall-bounded fluid flows^1–4^.

Using the porous media in the internal flows meets some of the mentioned theories for increasing the heat transfer rate in the internal flows. The heat transfer enhancement of the heated channels filled by the metallic porous media has been confirmed by several studies^5–8^. For example, Azizifar et al.^7,8^ discovered more than five times heat transfer improvement by using porous media. Open-celled metal foams, as a passive technique for heat transfer improvement, could adequately increase heat transfer area without any volume increment^9^. Moreover, they include other thermal benefits like robust flow-mixing ability, high solid thermal conductivity, low-density. Hence, it is effective in compact heat sinks and heat exchangers^10,11^, solar collectors^12^, efficient combustors^13^, and chemical reformers^14^.

Despite the attractive thermal performances for metal foams, further improvements are required for heat transfer in metal foams through other assistant approaches in some heat exchangers and electronics with high heat transfer rates. The addition of nanoparticles into the base working fluid, known as nanofluid, is such a direct technique for increasing the fluid thermal conductivity. The heat transfer enhancement of the nanofluid flow has been verified by studies^15–17^ in different types of applications. For example, Al-Hossainy and Eid^18^ found a significant heat absorption by using nanofluid in a cooling system of a solar cell. Since the heat transfer increment of the nanofluid was discovered by studies, some works were dedicated to the nanofluids’ heat transfer features through porous media^5,19–23^. The nanofluid, in turn, could improve the heat transfer in the internal flows. The insertion of porous media in channels has shown an additional improvement. So, the convective flow of nanofluids in metal foam (porous) tubes is an attractive research area for heat transfer application. Heat and mass transfer of nanofluids through the porous media was numerically investigated by Agarwal and Bhadauria^19^. In another study, the heat transfer enhancement of a porous channel by the dispersion of the nanofluid in the conventional fluid was reported^24^. Amani et al.^25^ also found the Nusselt number increment for the convective flow of nanofluids in a porous pipe.

Computational fluid dynamics (CFD) modeling of the nanofluid flows in porous media has been commonly used by studies^26,27^ for the prediction of the flow characteristics. However, the CFD method, alone, is not a perfect way, especially in complicated cases (e.g. large scale 3 dimension geometries, complex shapes, turbulent flows, multiphase flows, etc.). The complicated cases require expensive computational facilities; consume a lot of time and effort, etc. The application of artificial intelligence (AI) algorithms has been recently shown by some publications^28–33^. The artificial intelligence algorithm can optimize the CFD approach. Once the governing equations (i.e., mass, momentum, energy, etc.) are discretized and solved by the CFD methods for several specific boundary conditions, the pattern of the CFD predictions can be found by the machine learning of the AI. Then, there is no need for CFD anymore. The new predictions of any thermal and hydraulic parameters could be done for new boundary conditions or more mesh density. Using the AI algorithm makes us needless from solving the complicated governing equations. The fuzzy inference has been previously combined with the algorithms of the adaptive network and the ant colony to learn the CFD modeling solutions of the bubbly flow in column reactors^34,35^. But, the deep investigations about the other AI algorithms and the importance of the tuning parameters are absent in the literature. In addition, the ability of mesh increment in AI algorithms has not been shown in the literature. This study presents the ability of the AI algorithm for mesh increment. For the first time, the genetic algorithm in combination with the fuzzy inference is adopted to learn the CFD results of the nanofluid flow in a heated porous tube. The parameters of the genetic algorithm and fuzzy inference system for the most accurate predictions of the nanofluid velocity are evaluated.

## Methodology

### Geometry and boundary conditions

Turbulent convective flow of the water-based copper nanofluid in a porous tube is selected for the simulation. The pipe diameter and the length are 10 mm and 1 m, respectively. The pipe is under a constant heat flux of 115 kw/m^2^. The pipe is filled by aluminum porous media with a porosity of 0.8 and a pore’s density of 10 PPI (pores per inch). The copper nanoparticles’ concentration is 2% in the whole volume of water. The nanofluid is supposed to enter the pipe with the velocity and the temperature of 0.91 m/s and 300 °K, respectively.

### CFD approach

Considering steady-state turbulent flow for an incompressible Newtonian fluid, the governing equations are given as follows^36^:

Mass:

1 $$\nabla \cdot \left({\rho }_{eff,nf}U\right)=0$$

Momentum:

2 $$\frac{1}{{\gamma }^{2}}\nabla \cdot \left({\rho }_{eff,nf}\overrightarrow{U}\overrightarrow{U}\right)=-\nabla p+\frac{1}{\gamma }\nabla \left[{\mu }_{eff,nf}^{e}\left(\nabla \overrightarrow{U}+{\left(\nabla \overrightarrow{U}\right)}^{T}\right)\right]-\frac{{\mu }_{eff,nf}}{\beta }\overrightarrow{U}-\frac{{\gamma {C}_{in}\rho }_{eff,nf}}{\sqrt{\beta }}\left|U\right|\overrightarrow{U}$$

Energy:

3 $$\nabla \cdot \left({\rho }_{eff,nf}U{C}_{p.eff,nf}T\right)=\nabla \cdot \left(\gamma {k}_{tot}\nabla T-{\gamma ({\rho C}_{p})}_{eff,nf}\overline{UT}\right)$$

where γ, *C*_*in*_, and *β* are the porosity, the inertia coefficient, and the permeability respectively and these parameters are calculated as follows^27,37–39^:

4 $$\beta =0.00073{d}_{pore}^{2}{\left(1-\gamma \right)}^{-0.224}{\left(\frac{{d}_{cell}}{{d}_{pore}}\right)}^{-1.11}$$

5 $${C}_{in}=0.00212{\left(1-\gamma \right)}^{-0.132}{\left(\frac{{d}_{cell}}{{d}_{pore}}\right)}^{-1.63}$$

6 $${d}_{pore}=0.0254(m)/10(PPI)$$

7 $${d}_{cell}=1.18{d}_{pore}\sqrt{\frac{1-\gamma }{3\pi }}\left(\frac{1}{1-{e}^{\frac{-\left(1-\gamma \right)}{0.04}}}\right)$$

Aluminum porous media with a porosity of 0.8 and 10 PPI pore density is considered for this investigation. The total thermal conductivity is comprised of the effective nanofluid thermal conductivity and that of the solid matrix of the aluminum metal porous^27,39^:

8 $${k}_{tot}=(1-\gamma ){k}_{Al}+\gamma {k}_{eff,nf}$$

The standard $k-\varepsilon$ model is adopted for considering the turbulence effect^40–42^.

9 $$ \nabla \cdot (\rho_{eff,nf} kU) = \nabla \cdot \left[ {\left( {\frac{{\mu_{t} }}{{\sigma_{k} }}} \right)\nabla (k)} \right] + G_{k} - \rho_{eff,nf} \varepsilon $$

10 $$ \nabla \cdot (\rho_{eff,nf} \varepsilon U) = \nabla \cdot \left[ {\frac{{\mu_{t} }}{{\sigma_{\varepsilon } }}\nabla \varepsilon } \right] + \frac{\varepsilon }{k}(C_{1\varepsilon } G_{k} - C_{2\varepsilon } \rho_{eff} \varepsilon ) $$

$$ \begin{aligned} & G_{k} = \mu_{t} (\nabla U + (\nabla U)^{T} ), \, \mu_{t} = \rho_{eff,nf} C_{\mu } \frac{{k^{2} }}{\varepsilon } \\ & C_{\mu } = 0.09,\sigma_{k} = 1.00,\sigma_{\varepsilon } = 1.30,C_{1\varepsilon } = 1.44,C_{2\varepsilon } = 1.92 \\ \end{aligned} $$

### Water-based copper properties

For viscosity and conductivity of water-based copper nanofluid, these values could be found in the experimental study of Xuan and Li^43^ for the nanoparticle volume fraction of 2% (k_eff,nf_ = 0.702 W/m k and ν = 1.125 × 10^–6^). The following equations are also used for calculating the effective density and the specific heat at constant pressure:

12 $$ \rho_{eff,nf} = (1 - \alpha )\rho_{water} + \alpha \rho_{p,Cu} $$

13 $$ c_{p,eff,nf} = \frac{{(1 - \alpha )(\rho c_{p} )_{water} + \varphi (\rho c_{p} )_{p,Cu} }}{{(1 - \alpha )\rho_{water} + \varphi \rho_{p,Cu} }} $$

### CFD verification

**Figure 1:**
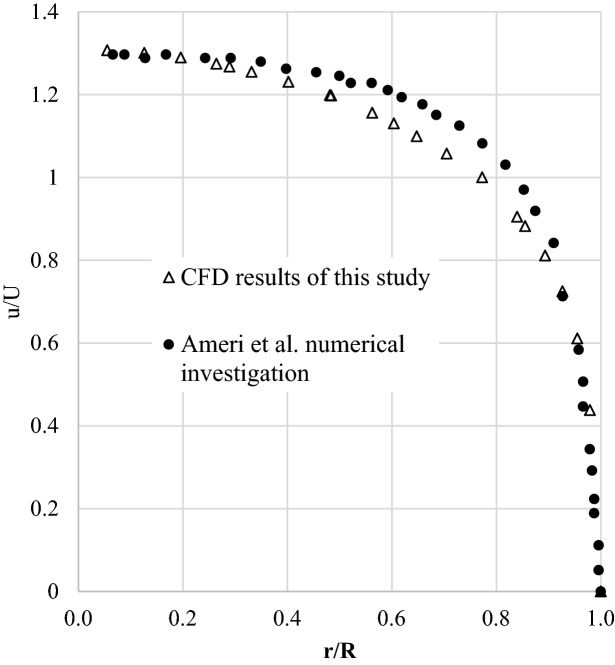
Comparison of velocity profile predicted by CFD from this study and that from Ameri et al.^26^.

The experimental data is not available for the turbulent convective flow of the nanofluid water-based in a porous tube. So, the average Nusselt number (Nu_ave_) from this study is compared with the experimental results of Xuan and Li for turbulent convective flow of water-based copper nanofluid in a simple heated tube. According to experimental results, for the 2% vol. water-based copper nanofluid in the simple tube the Nu_ave_ is around 80, while this value is enhanced by more than three times to around 288 in the porous tube. In addition, the velocity profile of the CFD simulation from this study is compared with that from the numerical investigation of Ameri et al.^26^ for water-based-Fe_3_O_4_ nanofluid flow in a heated porous tube (Fig. 1).

### Genetic algorithm (GA)

A genetic algorithm is initiated with some preliminary solutions (chromosomes), known as a population, which is then evolved into various populations for numerous (normally hundreds of) iterations. Ultimately, the algorithm returns the best member of the population as the solution to the problem. The evolution procedure proceeds as follows for every iteration or generation. Choosing two members of the population is performed in terms of some probability distribution. Then, these two members are integrated via a crossover operator for producing an offspring. This offspring is modified with a low probability, then via a mutation operator for introducing unknown search space to the population and improving the population’s diversity (the degree of difference amongst chromosomes in the population). The offspring is examined to check its suitability for the population. When it is proper, a replacement outline is utilized for selecting a member of the population and replacing it with the novel offspring. Now, a novel population exists and the evolution procedure is repeated till meeting definite conditions, for instance, followed by a fixed number of generations. Only one offspring is generated by the present genetic algorithm per generation. This is known as steady-state genetic algorithm^44,45^, versus a generational genetic algorithm replacing the entire population or the population’s large subset per generation. Adding a local enhancement heuristic, normally after mutation, it is called hybridized and a GA with this outline is known as a hybrid GA. We will provide a steady-state hybrid GA for the graph partitioning problem.

### Fuzzy inference system (FIS)

The fuzzy inference method (FIS) is a widely used calculating mechanism for fuzzy reasoning, fuzzy set theory, and if–then rules. Automatic monitoring, computer vision, data classification, expert systems, and decision analysis all benefitted from it. There are three types of fuzzy logic in which Takagi–Sugeno’s if–then rules are implemented in a FIS framework^46^. The incoming signals are multiplied using the AND law. In this case, the i-th rule function can be expressed as follows:

14 $${w}_{i}={\mu }_{Ai}\left(X\right) {\mu }_{Bi}\left(Y\right)$$

In the equation above, ${w}_{i}$ denotes the firing strength; besides, ${\mu }_{Ai}$, ${\mu }_{Bi}$ and ${\mu }_{Ci}$ are respectively the incoming signals from the MFs that have run on inputs, x-coordinate (X) and y-coordinate (Y).

The relative firing strength value is defined for each rule. Accordingly, the overall firing strengths value for all rules can be defined as follows:

15 $$\overline{{w }_{i}}=\frac{{w}_{i}}{\sum \left({w}_{i}\right)}$$

In the equation above, $\overline{{w }_{i}}$ represents the normalized firing strength. The function of a consequent Takagi–Sugeno’s if–then rule is then applied^46^.

Therefore, the node function can be expressed as follows:

16 $$\overline{{w }_{i}}{f}_{i}=\overline{{w }_{i}}({p}_{i}X+{q}_{i}Y+{s}_{i})$$

In the above equation, ${p}_{i}$, ${q}_{i}$, and ${s}_{i}$ are if–then rules’ parameters, referred to as consequent parameters. The output of the model can be achieved by integrating the incoming signals, which represents the result of estimation. These parameters are then updated by employing a hybrid learning algorithm. The gradient descent and least-squares estimation (LSE) methods are utilized to respectively update MF and consequent parameters.

## Results and discussion

The turbulent convective flow of the water-based copper nanofluid in a porous tube is modeled by the CFD. For the boundary conditions, the inlet velocity and temperature of the nanofluid are considered constant, while the tube wall is under constant heat flux.

**Figure 2:**
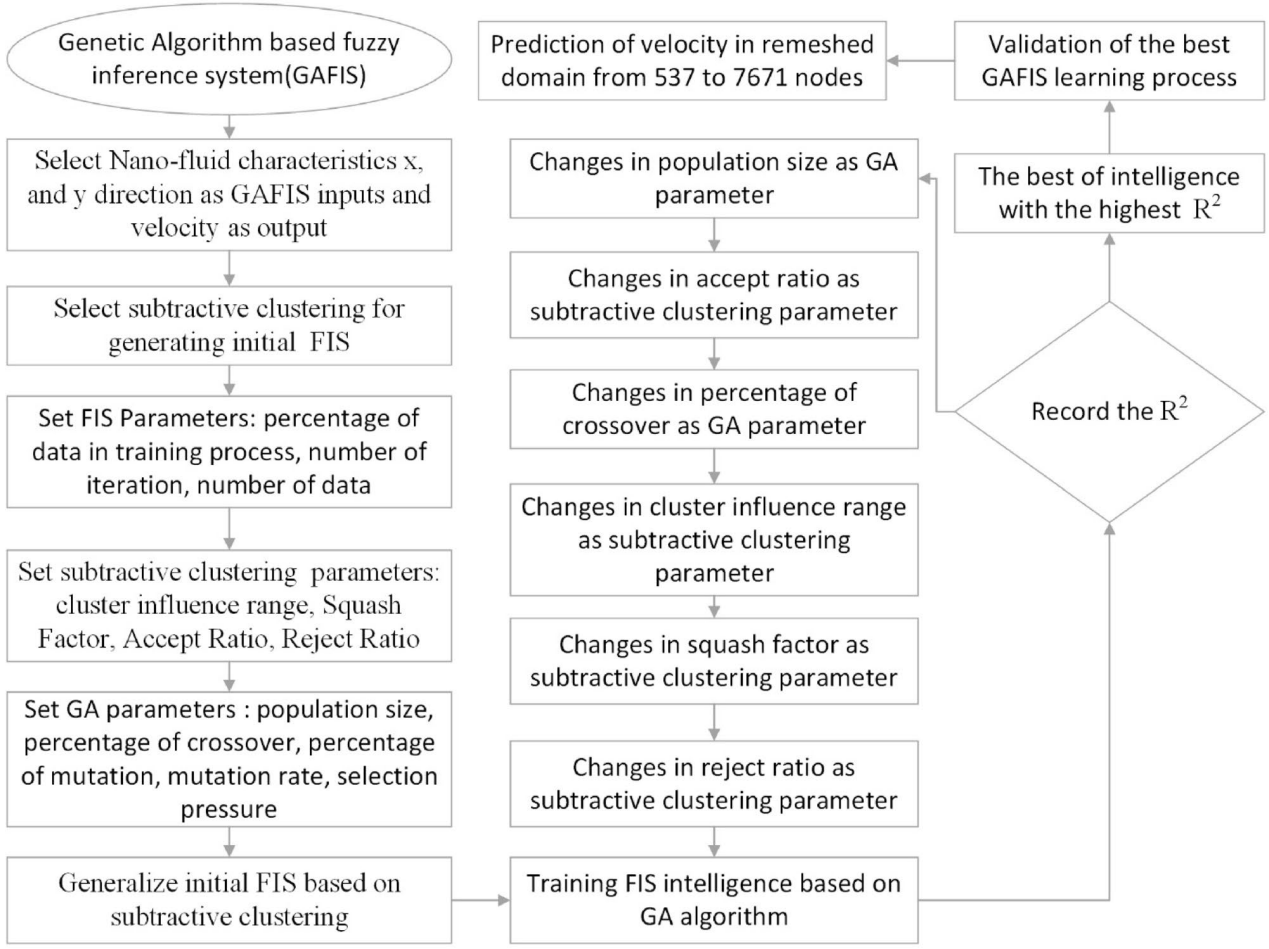
schematic of GAFIS method.

The nanoparticles distribution is supposed to be homogenous and as a result, the single-phase model is considered for the mixture of the solid nanoparticles and the based fluid. The finite volume method (FVM) is used as the discretization. The ability of mesh increment of the AI algorithm in the prediction of velocity, for nanofluid flow in a porous tube is investigated. Increasing the mesh density causes a significant increase in the computational efforts by the CFD. But this is done more straightforward by the AI algorithm. The AI algorithm predicts the results based on the pattern of the CFD results. This study tries to present the efficiency of the genetic algorithm in a combination with fuzzy inference. Figure 2 describes the whole steps from the setup of the GAFIS to the predictions of the velocity. The x and y coordinates of the nodes on the cross-section plate (0.4 m from the inlet) are the inputs, and the velocity of the nanofluid is the output. Totally, 537 CFD data are available and 80% of them are used for training during 700 iterations. Since subtractive clustering is used for inertia FIS, the parameters such as cluster influence range (CIR), squash factor (SF), accept ratio (AR), and reject ratio (RR) must be determined.

In this study, an analysis is done on adjusting the proper values of all subtractive clustering parameters for the best intelligence achievement.

Next step, the genetic algorithm (GA) parameters, including the population size, the percent of mutation, the mutation rate, the percentage of crossover, and the selection pressure, are defined.

Another sensitivity test is also done for adjustment of the population size and the percentage of crossover, while the effects of the remaining parameters of the GA are neglected in this study.

**Figure 3:**
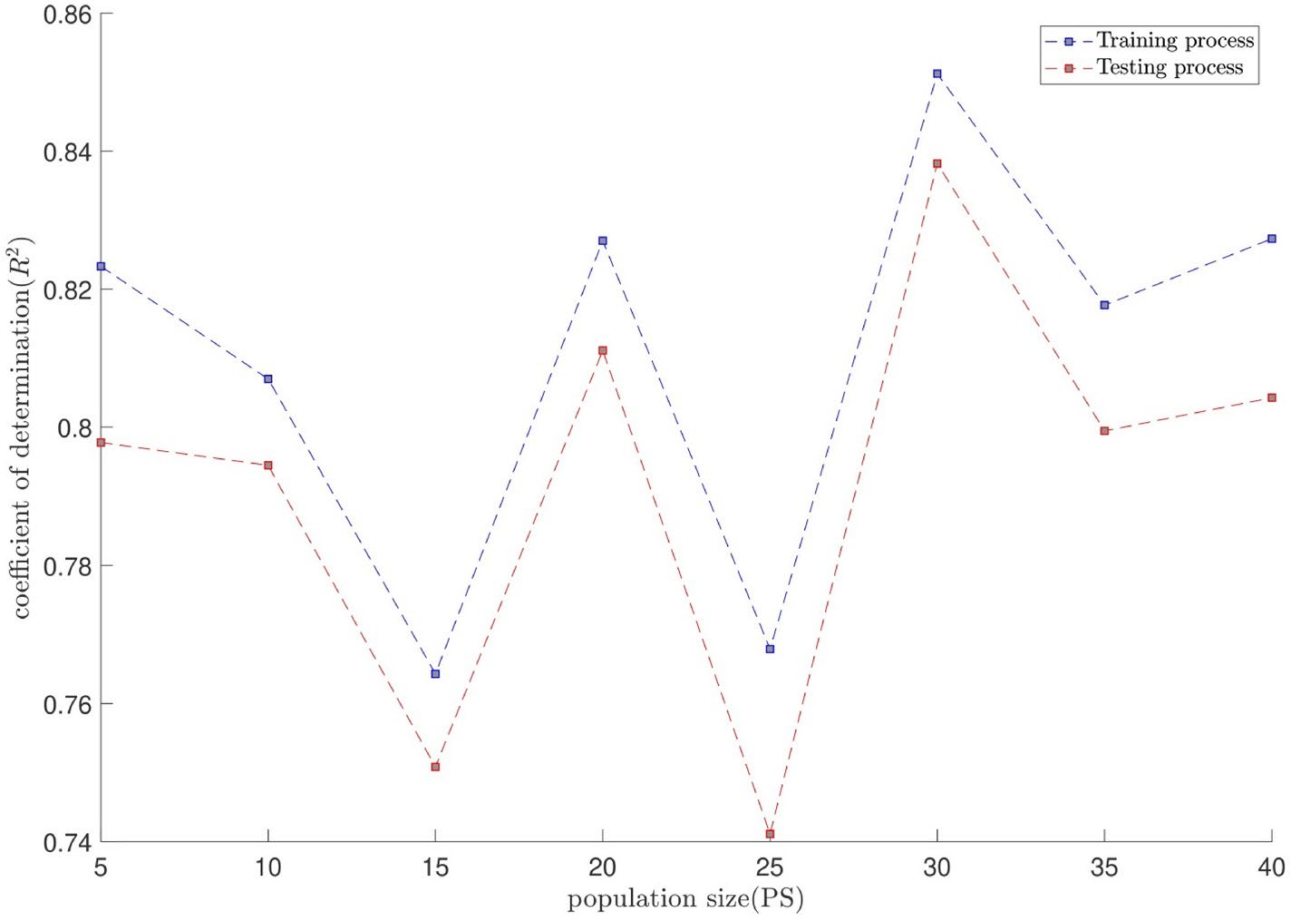
GAFIS method learning processes with changing in population size (P.S).

**Figure 4:**
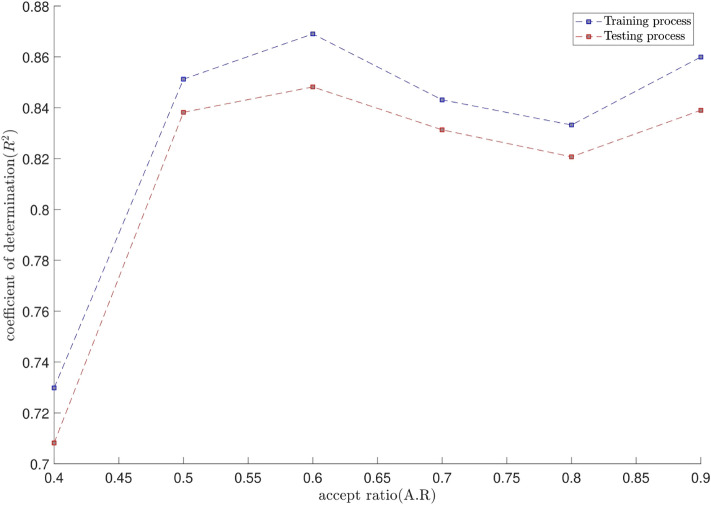
GAFIS method learning processes with changing in accept ratio (A.R).

**Figure 5:**
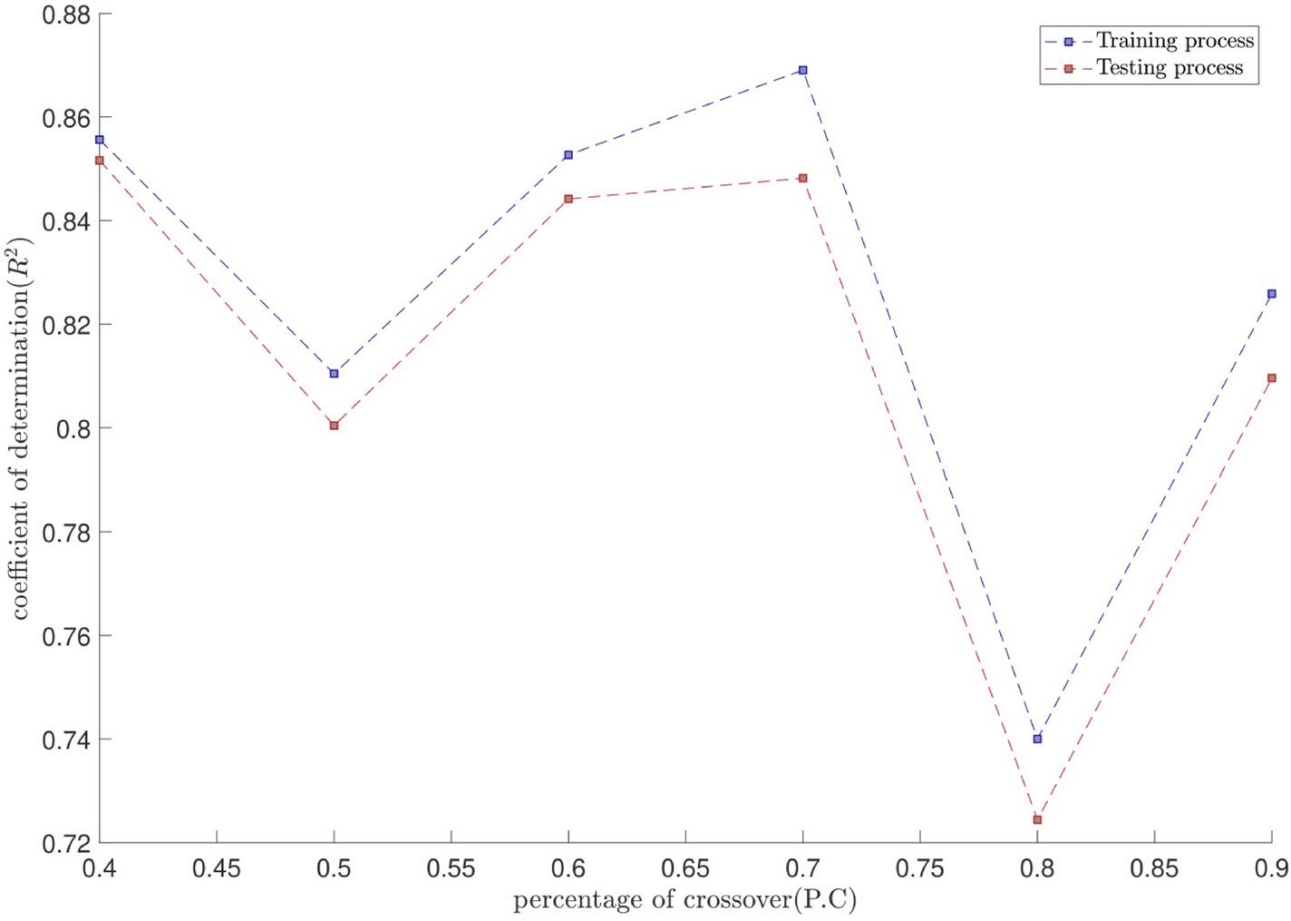
GAFIS method learning processes with changing in percentage of crossover (P.C).

**Figure 6:**
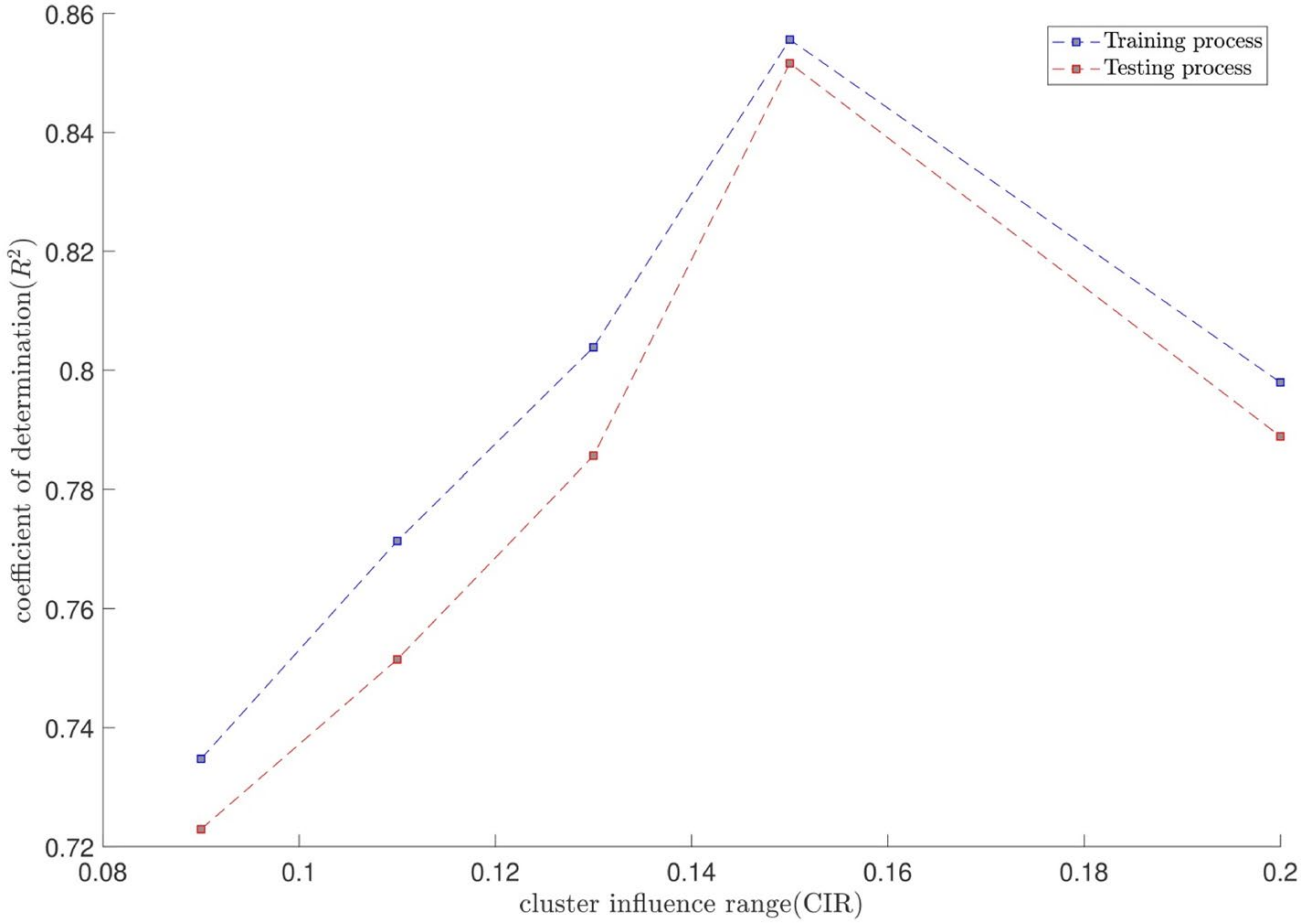
GAFIS method learning processes with changing in cluster influence range (CIR).

**Figure 7:**
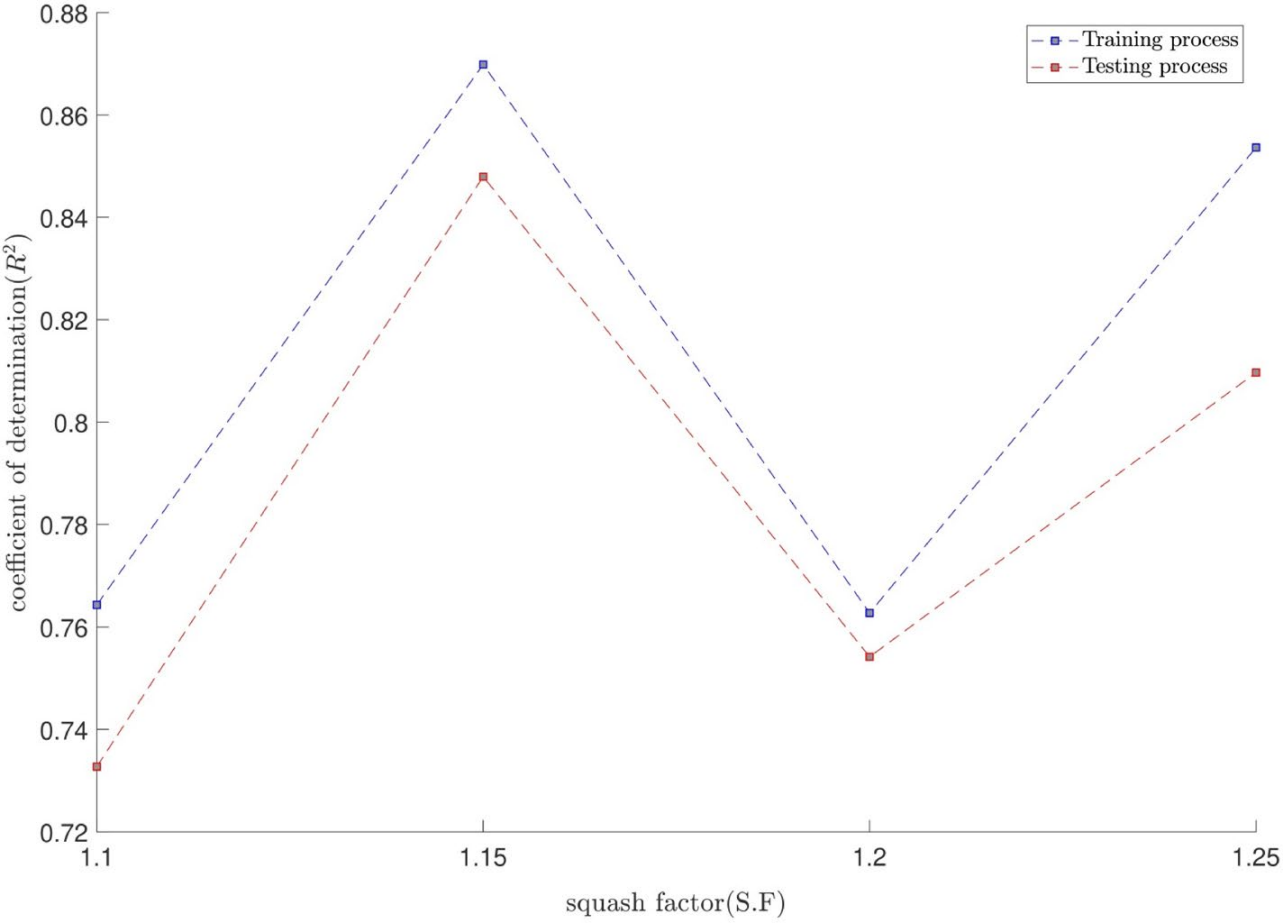
GAFIS method learning processes with changing in squash factor (S.F).

**Figure 8:**
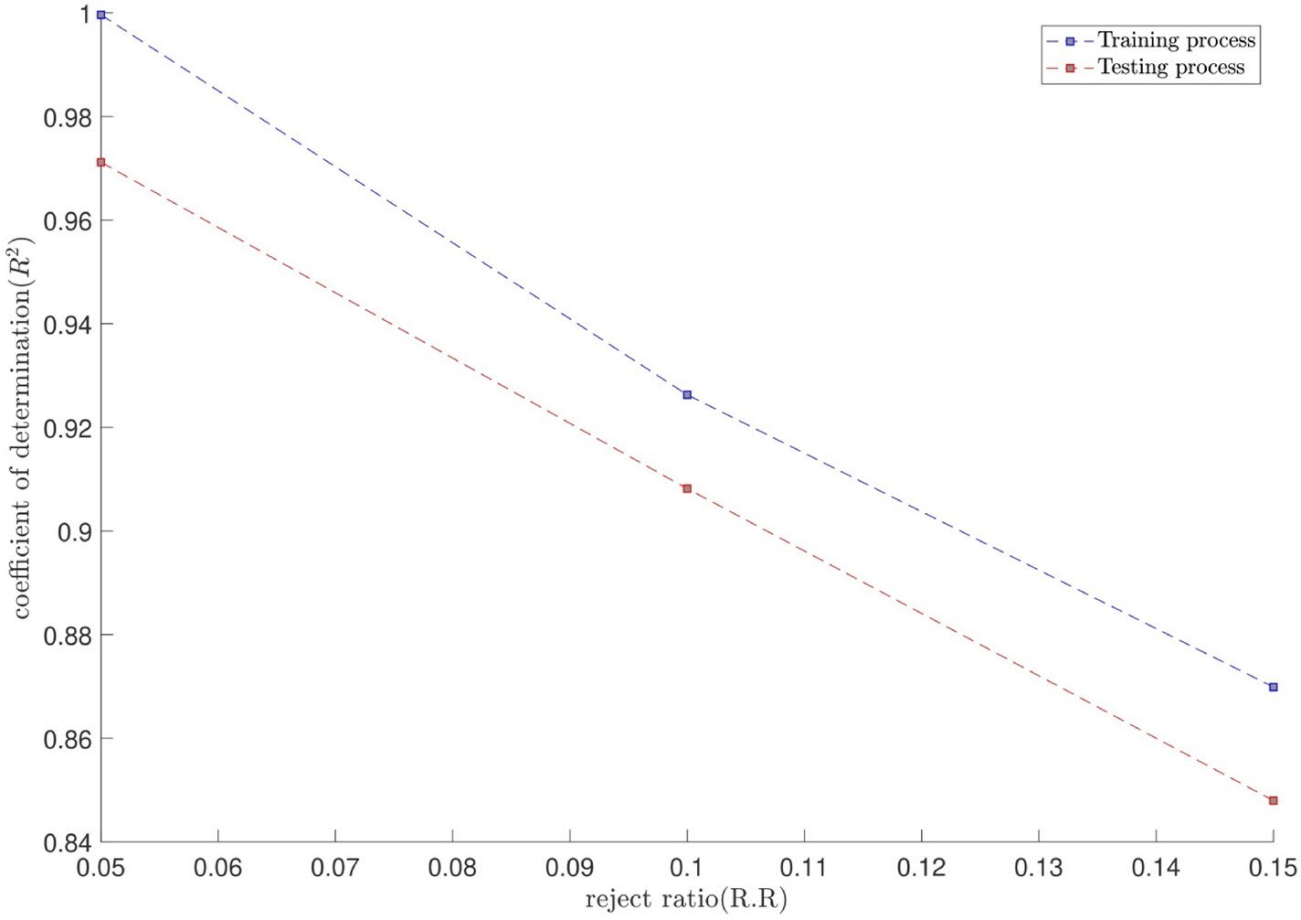
GAFIS method learning processes with changing in reject ratio (R.R).

**Figure 9:**
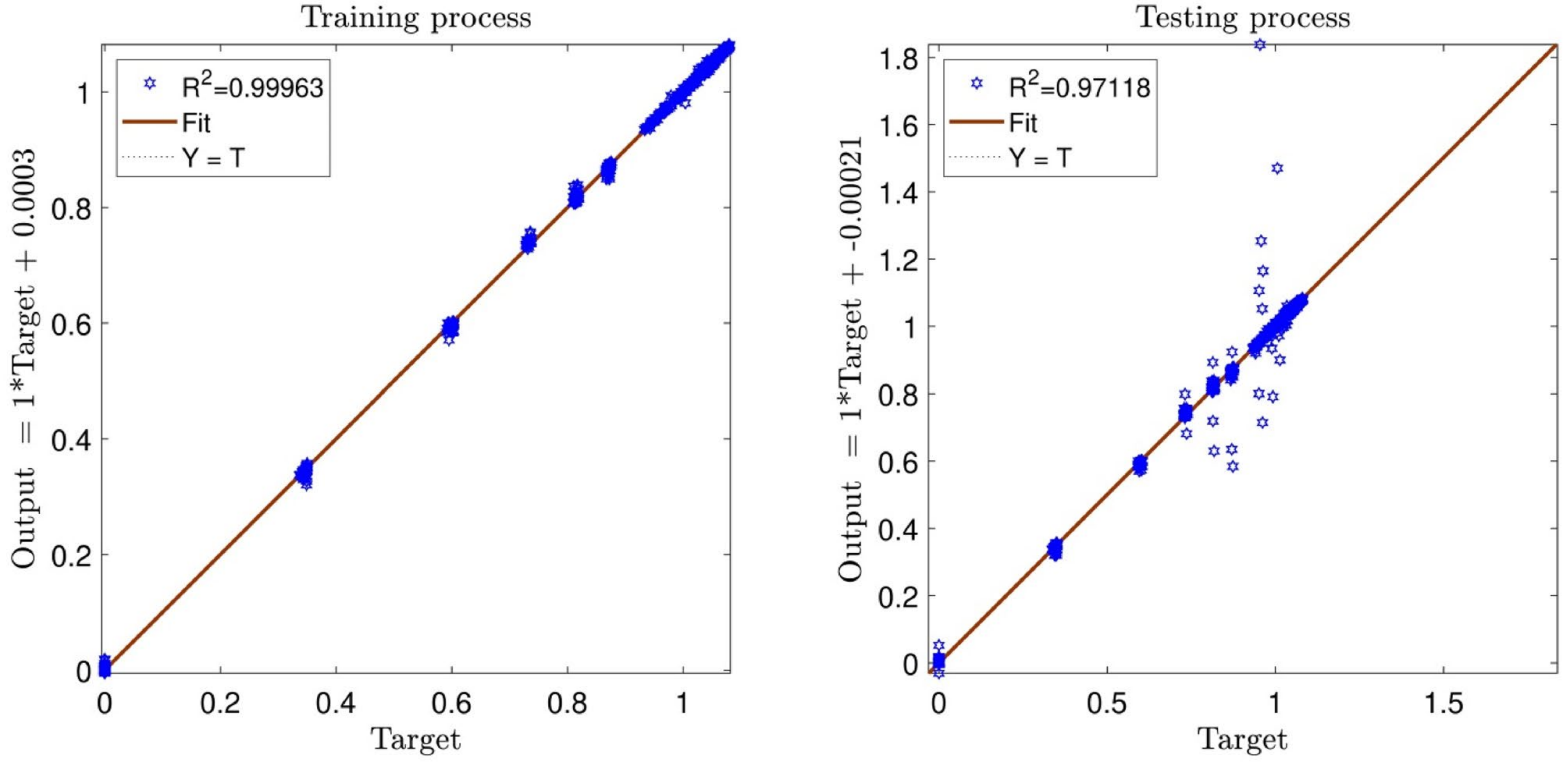
Determination of coefficient in the best GAFIS intelligence when P.S = 30, A.R = 0.6, P.C = 0.4, CIR = 0.15, S.F = 1.15, R.R = 0.05.

**Figure 10:**
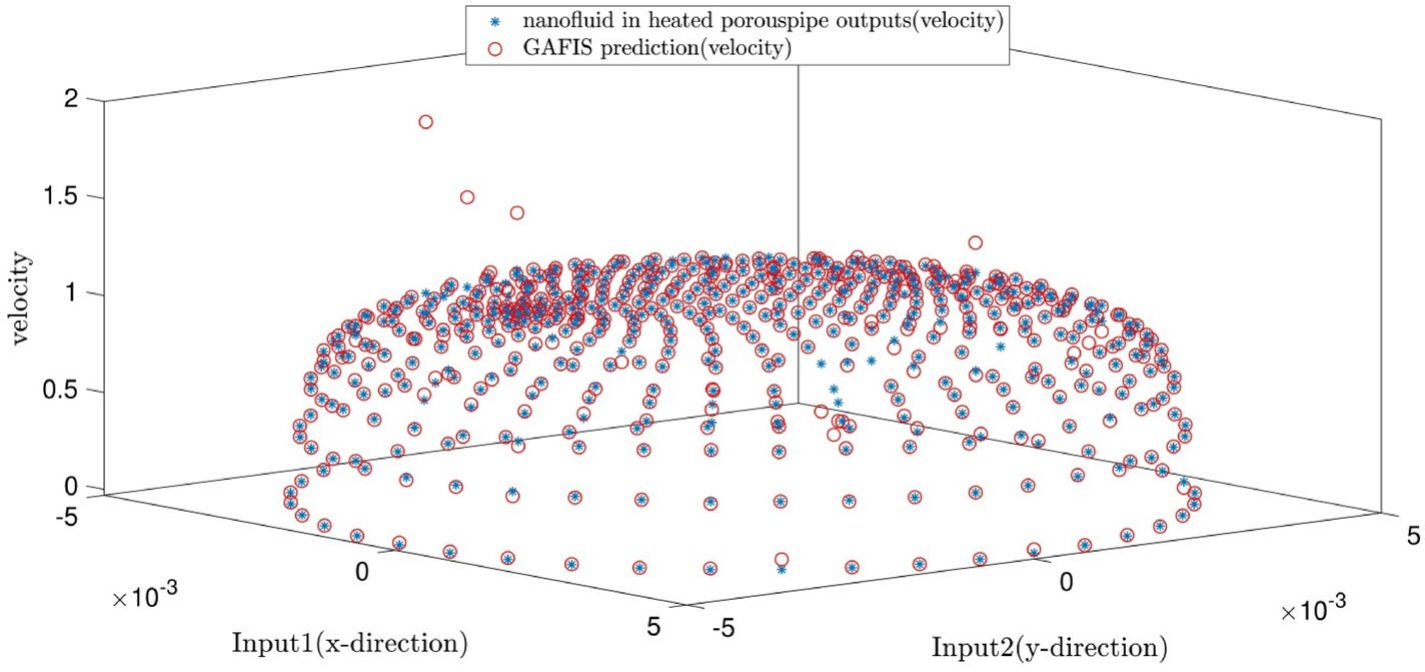
Validation of learning process with comparison of prediction data and CFD results.

**Figure 11:**
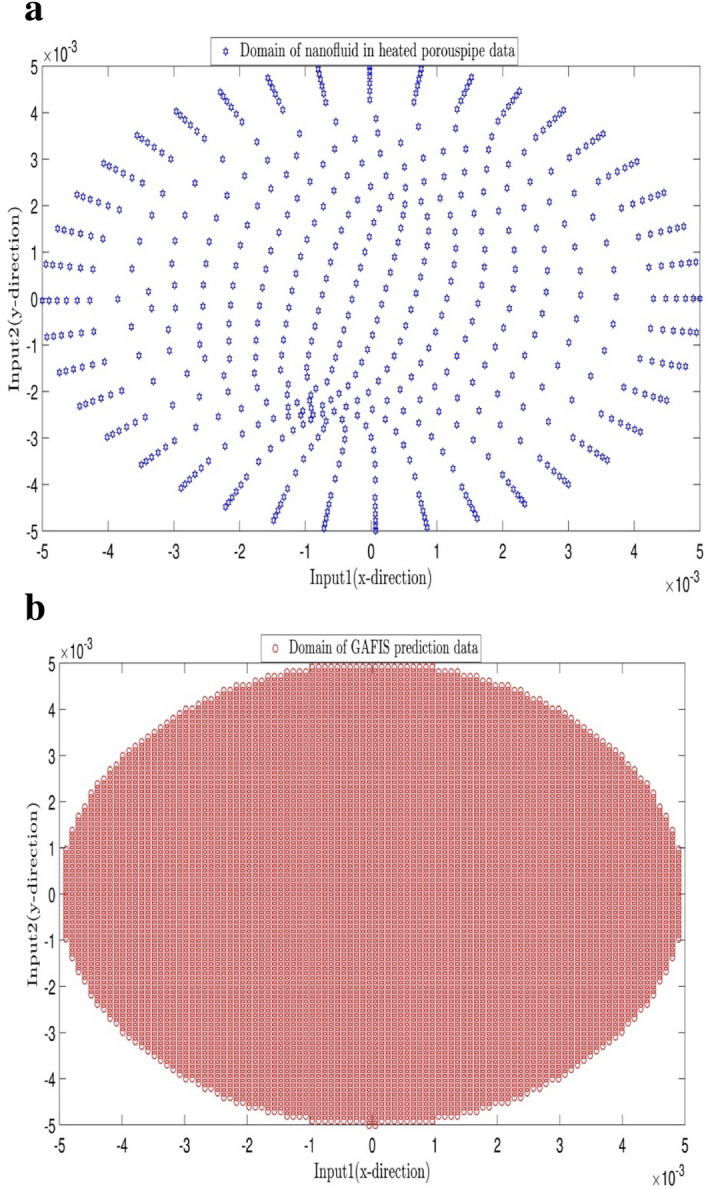
(**a**) CFD nodes which is considered in learning process (537 nodes). (**b**) Remeshed domain from 537 to 7671 nodes for prediction.

**Figure 12:**
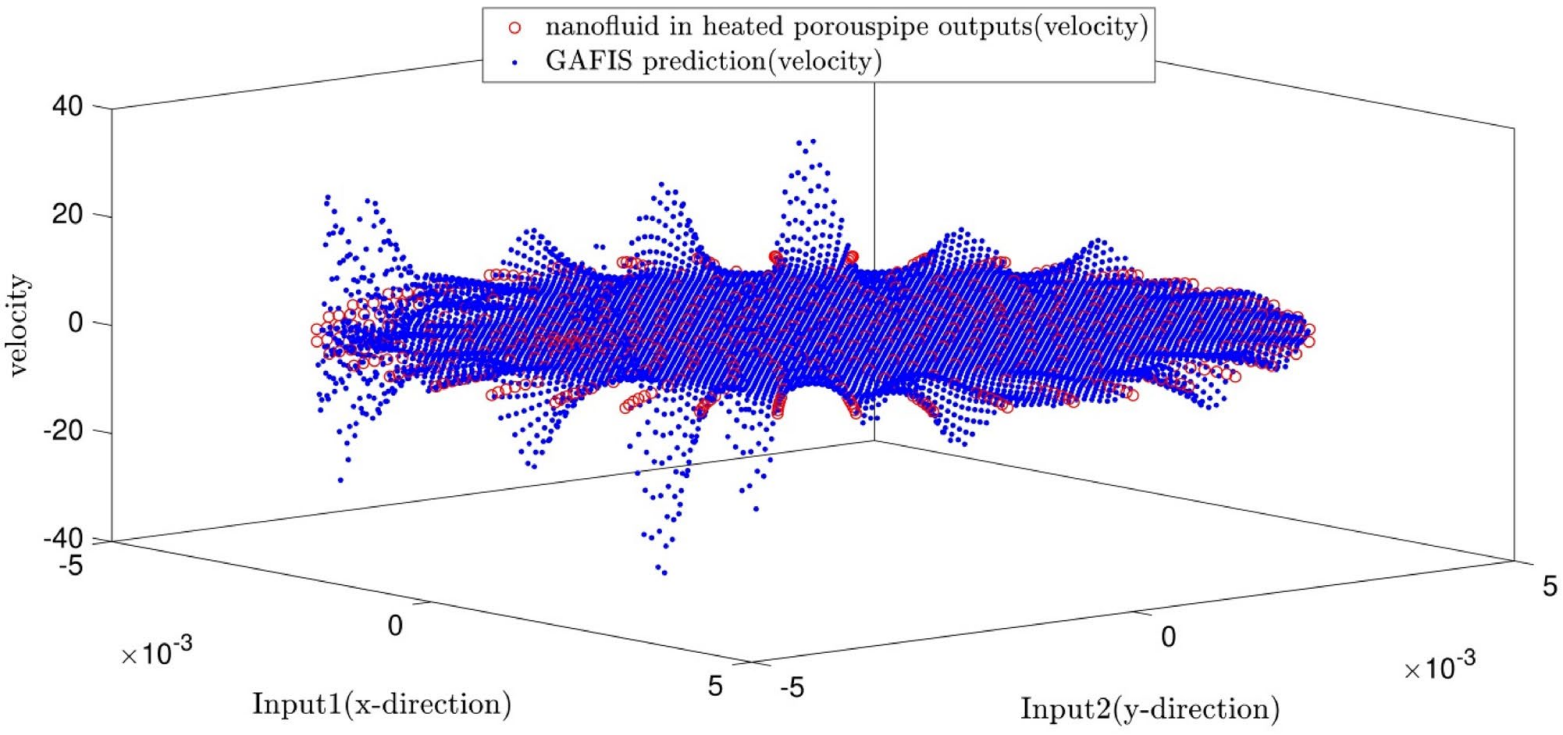
Prediction of velocity in remeshed domain and its comparison with velocity as CFD output.

The tuning process for all parameters is stopped, once the highest level of intelligence is obtained. The FIS (fuzzy inference system) trains the CFD data based on GA (genetic algorithm); the coefficient of determination is recorded for different values of PS, AR, CIR, SF, and RR. In each step of the sensitivity test, the corresponding value of the parameter to the highest R^2^ is adopted. The maximum value of R^2^ means that the best intelligence of the GAFIS. The GAFIS prediction of the nanofluid velocity is validated by the CFD results. After finding the best intelligence, the mesh increment is done by the GAFIS. Figures 3, 4, 5, 6, 7 and 8 illustrate the change of R^2^ by adjusting the mentioned tuning parameters. The results released that the maximum R^2^ values are related to the PS of 30, the AR of 0.6, the PC of 0.4, CIR of 0.15, the SF 1.15, and the RR of 0.05. According to Fig. 9, once all the best tuning parameters are considered together in the GAFIS, the R^2^ is equal to 0.97 for the testing process. The GAFIS prediction of the nanofluid velocity is in great agreement with the CFD, as shown in Fig. 10. Figure 11 shows the number of nodes based on the meshing process in the CFD (Fig. 11a) and the increase of the number of nodes from 537 to 7671 in the GAFIS (Fig. 11b). Although such mesh increment in the CFD requires a high capacity computer (high RAM, graphic card, etc.), this can be done with lower computational infrastructures in the GAFIS. Figure 12 illustrates the predictions of the GAFIS for the new nodes. The new predictions of the GAFIS cover all CFD results. A few faults are seen near the wall of the pipe. This means maybe more CFD data are needed for finding a precise pattern of the fluid velocity near the wall (hydrodynamic boundary layer). Further investigations are suggested for the future to consider the wall effect in artificial intelligence predictions of the CFD results.

## Conclusions

This study tried to establish an auxiliary approach to remove the barriers of the mesh increment in the CFD modeling. The mesh increment in the CFD approach could be time-wasting and overpriced, especially in large scales, turbulent flows, complex geometries, etc. So, the 3-dimension CFD modeling of turbulent convective flow of the water-based copper nanofluid in a porous tube was selected as a case study. The genetic algorithm in a combination with fuzzy inference system (GAFIS) was used for learning the CFD results. The velocity profile of the nanofluid was adopted as a variable for assessing the performance of the GAFIS. It was supposed that for the intelligence condition, the GAFIS could find the pattern of the CFD results and predict the velocity precisely. After achieving the intelligence, the number of nodes could be increased by the GAFIS and the velocity could be found for more nodes without any CFD modeling. The tuning process was done for adjusting the parameters such as cluster influence range (CIR), squash factor(SF), accept ratio (AR) and reject ratio (RR), the population size (PS), and the percentage of crossover (PC). The coefficient of determination (R^2^) was recorded for different values of PS, AR, CIR, SF, and, PC, and RR. The value of R^2^ would be maximum for the best intelligence of the GAFIS. The GAFIS prediction of the nanofluid velocity was validated by the CFD results.

The main findings of this study are as follows.

- The most intelligent GAFIS is achieved for the PS of 30, the AR of 0.6, the PC of 0.4, CIR of 0.15, the SF 1.15, and the RR of 0.05.
- The coefficient of determination is 0.97 at the most intelligent condition.
- The GAFIS prediction of the nanofluid velocity was in great agreement with the CFD.
- The strong capability of GAFIS for capturing the CFD data and nodes increment was obtained. The nodes increment from 537 to 7671 was made by the GAFIS. The new predictions of the GAFIS precisely covered all CFD results.
- A few faults were found near the wall of the pipe. This could be justified by insufficient data learning near the wall. Further investigations are suggested for future studies to analyze the wall effect on artificial intelligence predictions.
